# Effect of Particle Size Distributions and Shapes on the Failure Behavior of Dry Coke Aggregates

**DOI:** 10.3390/ma14195558

**Published:** 2021-09-24

**Authors:** Alireza Sadeghi-Chahardeh, Roozbeh Mollaabbasi, Donald Picard, Seyed Mohammad Taghavi, Houshang Alamdari

**Affiliations:** 1 Aluminum Research Centre–REGAL, Mining, Material, and Metallurgy Engineering Department, Université Laval, 1065 Avenue de la Médecine, Québec, QC G1V 0A6, Canada; alireza.sadeghi-chahardeh.1@ulaval.ca (A.S.-C.); roozbeh.mollaabbasi.1@ulaval.ca (R.M.); 2 Eddyfi Technologies Company, 3425 Rue Pierre-Ardouin, Québec, QC G1P 0B3, Canada; dpicard@eddyfi.com; 3 Chemical Engineering Department, Université Laval, 1065 Avenue de la Médecine, Québec, QC G1V 0A6, Canada; seyed-mohammad.taghavi@gch.ulaval.ca

**Keywords:** failure analysis, particle size distribution, particle shape, rolling resistance, second-order work criterion, strain localization

## Abstract

Carbon anodes participate in chemical reactions to reduce alumina in the Hall–Héroult process, of which coke aggregates make up a major part. The failure analysis of coke aggregates not only leads to a better understanding of the deformation mechanisms of anode paste under compressive loading but also can identify potential causes of structural defects in carbon anodes, such as horizontal cracks. The coke aggregates are composed of particles with different size distributions and shapes, which may strongly affect the failure behavior of the anode during compaction. In this paper, the effects of particle size distributions and shapes on the mechanical behavior and the failure of coke aggregates are investigated using the discrete element method modeling technique. The numerical results reveal that, although the mechanical behavior of coke mixtures is generally dependent on larger particles, the presence of fine particles in the coke aggregates reduces fluctuations in the stress–strain diagram. In addition, the rolling resistance model is employed as a parameter representing the effect of particle shape. It is shown that the rolling resistance model can be an alternative to the overlapped spheres model, which has a higher computational cost than the rolling resistance model. The second-order work criterion is used to evaluate the stability of the coke aggregates, the results of which indicate that the addition of fine particles as well as increasing the rolling resistance between the particles increases the stability range of the coke aggregates. Moreover, by using the analysis of micro-strain contour evaluations during the compaction process, it is shown that, both by adding fine particles to the coke mixture and by increasing the rolling resistance between the particles, the possibility of creating a compression band in the coke aggregates is reduced. Since the presence of the compaction bands in the anode paste creates an area prone to horizontal crack generations, the results of this study could lead to the production of carbon anodes with fewer structural defects.

## 1. Introduction

In aluminum smelters, alumina is transformed into aluminum through the Hall–Héroult electrolysis process, in which carbon and electricity are consumed. The required carbon and electricity in this process are supplied by and through carbon anodes. The carbon anodes consist of about 85% dry aggregates (the mixture of coke particles and recycled butts) and 15% coal-tar-pitch (CTP), which are mixed at a temperature of about 158 °C to obtain an anode paste [1]. The anode paste is poured into a rigid mold and goes through vibro-compaction or the pressing process to obtain green anode blocks.

The green anode blocks are baked at 1100 °C for about 20 days to achieve suitable mechanical and electrical properties through carbonization of the CTP. The quality of the carbon anode affects both the carbon consumption and the amount of electricity required, and consequently it may have a significant impact on the total cost of the aluminum production [2].

A high-quality carbon anode has low electrical resistance, high mechanical strength, and homogeneity as well as low reactivity towards carbon dioxide and air. The presence of any defects in the carbon anode, such as internal and external cracks and density gradients, disrupts the cell stability and increases carbon consumption by reducing the mechanical strength and the electrical conductivity of the baked anodes [1].

The generation of cracks in the carbon anodes can be originated from many factors, the most important of which are as follows: thermal shock during installation of the anode into the electrolysis cell [3], a high temperature gradient inside the anode due to the high heating rate during the baking process [4], and strain localization during the compaction process [5]. The thermal shock induced by immersing the anode in the electrolysis cell can generate corner cracks [6].

If the heating rate during the baking process is high, it can generate a high-temperature gradient inside the anodes, which helps to create the tensile stresses needed to form vertical cracks [4]. In addition, during the compaction process, the compressive strain can accumulate in a narrow rectangular area, resulting in a localized band. Due to the viscoelastic properties of the carbon anode paste, the compression accumulated in these localized bands causes residual tensile stresses in the carbon anodes [5].

This phenomenon can form horizontal cracks around the stub-hole, where the compressive pressures appear to be higher than other parts of the carbon anode due to the stub-hole shape effect [7]. The study of how cracks are generated in the anode and the parameters affecting it, both experimentally and numerically, has limitations. The dependence of the properties of the anode materials on temperature and the need to keep the temperature constant during the compaction process [8], as well as the opacity of the anode components [9], make the experimental study of anode paste quite challenging. In addition, numerical methods based on continuum mechanics, such as finite element methods, suffer from the lack of constitutive equations that are capable of modeling the granular behavior of anode paste [10].

Sadeghi-Chahardeh et al. [5] showed that the discrete element method (DEM) can be employed not only to investigate the causes of cracks but also as a virtual laboratory to study the parameters of the anode materials (such as the shape of coke particles) and the process of carbon anode production (such as the strain rate) to achieve an optimal carbon anode. However, studying the determinants of horizontal cracks in the carbon anodes requires a more in-depth analysis of the distinctive behaviors of granular materials under compressive loading conditions.

It has been shown that, when a granular material is exposed to a compressive load, at the initial time, it is deformed homogeneously. However, after reaching to a critical stress state, it is no longer able to sustain any deviatoric load increments. At such a critical stress state, if an additional load is imposed, the state of the material changes suddenly with the occurrence of large deformations, cracks, fragmentation, etc. [11,12]. This phenomenon, which is associated with a state of transition from a quasi-static regime to a dynamical regime, is called failure [13].

Experimental observations have shown that two broad classes of failure modes take place in the granular materials, mainly due to some instabilities, such as force-chain buckling [14,15,16]. In some cases, failure occurs in the form of a chaotic and unstructured strain field. This mode of failure, which is homogeneous and without any persistent and observable pattern of strain concentration, is called “*diffuse failure mode*” [17,18].

On the other hand, the failure in the granular materials under loading may transpire in a heterogeneous deformation pattern, in which the strain is largely localized into a system of bands. This pattern of failure is called “*localized failure mode*” [19,20]. The localized failure, according to the loading path and its kinematic attributes, can occur in the form of shear, dilation, and compaction bands [21]. The shear bands are often formed by the shearing load, while the dilation and compaction bands are formed by the volumetric deformation and are characterized by local volume expansion and local volume reduction, respectively [21].

While the sufficient condition for occurrence of the diffuse failure mode is predicted by Hill’s second-order work criterion [17,22], the vanishing value of the determinant of the acoustic tensor [23], which is known as classical bifurcation analysis, is employed for finding the sufficient condition for the localized failure mode.

Borja [24] revealed that both localized and diffuse failures could be predicted through the classical bifurcation analysis. However, due to the lack of a reliable constitutive law for predicting the granular material behavior, this method suffers from an inherent mesh-dependency problem [5,25].

Nicot and Darve [26] showed that Hill’s second-order criterion could also meet the necessary conditions for the occurrence of a local failure, which is the vanishing of the determinant of acoustic tensor. As a result, it would be able to detect both the diffuse and the localized failure mode. The advantage of this criterion over the classical bifurcation analysis is that it does not necessarily require a constitutive law to predict the sufficient condition of failure in granular materials [27].

As failure is recognized as a mechanism for defect formation in granular materials, an attentive examination of failure can shed light on the hidden facts about the origin of carbon anode cracking [21,28]. Sadeghi et al. [5] explained the formation of horizontal cracks in the carbon anodes by comparing them with the horizontal cracks in rock and sand mechanics and using DEM simulation of the coke aggregates. Accordingly, the strain localization bands, which are known as the main cause of defects in geology [29], are formed inside the carbon anodes during the compaction process.

These localized bands, which initially appear as shear bands, become compaction bands in the stub-hole areas, where exposed to a greater deformation than the other areas/regions. Although they showed how compaction bands are formed in the coke aggregates of carbon anodes (via employing DEM simulations), they did not examine the effects of particle size distribution and shape on the formation of the localized bands. Hence, investigating the role of size distribution and shape of particles on the failure behavior of coke aggregates would be valuable for a more accurate justification of the cracks in the carbon anodes.

The investigation of the particle size distribution effect on the mechanical behavior of granular materials has been the focus of researchers since the 1950s. The influence of the large particles on increasing the shear strength of granular materials was examined by Holtz and Gibbs [30] using triaxial tests on specimens of gravel and sand in various ratios. By studying clay samples containing different proportions of gravel, Shakoor and Cook [31] revealed that, with increasing sand content, the shear strength increases and the apparent adhesion decreases.

Ng [32] investigated the contribution of small and large particles in the mechanical behavior of granular materials by performing two-dimensional discrete element modeling for samples with two different particle sizes. He determined two thresholds for the fine particle contents, ${f\;c}_{l}$, ${f\;c}_{m}$. If the content of fine particles is less than ${f\;c}_{l}$, the contribution of small particles to the mechanical behavior of the mixture becomes negligible, while for the content of fine particles beyond ${f\;c}_{m}$, the mechanical contribution of large particles will be trivial.

Using a simplified packing structure model, Ueda et al. [33] were able to provide approximate values for the thresholds and evaluate their accuracy to describe the evaluation of shear strength transfer. They found that the mechanical contribution of small particles could not be ignored, even if their amount was small. Whereas, if the volume fraction of large particles is 2 or less with respect to small particles, the contribution of large particles to the mechanical behavior of granular materials is negligible.

In addition, by using the direct shear test on the sand-gravel mixtures, Simoni and Houlsby [34] demonstrated that the addition of gravel to a mixture, even at a low fraction (less than 20%), causes the mixture to have a higher shear strength and maximum dilatation rate than do mixtures of pure sand at the same density. Amirpour et al. [35], by conducting conventional drained triaxial compression tests on the different grain-size distribution samples, showed that the shear strength and volumetric response of samples were significantly affected by the variation of the average particle size.

Zhou et al. [36] explored the effect of different fine particle contents on the undrained behavior of granular materials using the DEM. They found that increasing the fine particle content to a certain value (approximately 10%) can change the material response from strain hardening to liquefaction.

Jiang et al. [37] numerically investigated the effect of particle size distribution on the granular material behavior in a triaxial test. They showed that specimens with narrower particle size distributions experienced higher shear strength and dilatation than those with wider particle size distributions. Moreover, they also attributed the strength of materials in the critical region to the shape of the particles rather than to the size distribution of the particles. Although extensive research has been conducted to investigate the effects of particle size distribution on the mechanical behavior of granular materials, especially in soil mechanics, the role of particle size in the failure of the granular materials is still debatable.

The mechanical behavior of granular systems is strongly influenced by the particle shape, which is known as the primary characteristic of granular materials [38,39]. Many researchers have been able to discover the effects of particle shape on the behavior of granular materials through extensive experimental and numerical research. Cho et al. [40] by conducting systematic experimental investigations on soil, showed that the packing density of a granular material depends on the sphericity, roundness, and smoothness of the particles and as the particle shape moves away from the regularity state (decrease sphericity and/or roundness), the difference between the minimum and maximum of the void ratio and compressibility under the zero lateral strain loading increases, while the small-strain stiffness decreases.

Using the direct shear test on the sand mixtures, Yagiz [41] concluded that the angular gravel creates higher volume friction angles and makes it difficult for the grains to slide on each other. By using the direct shear tests on the specimens with a wide range of particle size and shape, Li [39] indicated that the volume friction angle of the soil mixtures increases with decreasing the sphericity and smoothness of particles.

Yang and Luo [42] investigated the effect of particle shape on the critical state of a granular material. They used mixtures of spherical glass beads and crushed angular glass beads with uniform quartz sand as specimens and a robust laser scanning technology to make objective and accurate measurements of the particle shape. They showed that, as the particles’ regularity (sphericity and roundness) increases, the rotation of the particles increases, and consequently the specimen tends to be more susceptible to liquefaction.

As the experimental observations have limitations for understanding the effect of particle shape on the mechanical behavior of granular materials, such as the non-reproducibility of granular material samples, thanks to advances in computational devices in recent decades, many researchers have used the discrete element methods (DEMs) to study the particle shape effects on the behavior of granular materials.

As in DEM, when the intrinsic discrete nature of granular materials is considered, it can be employed as a powerful tool to investigate the behavior of granular materials in the state of failure [43,44]. In the original DEM, for the sake of enhancing the simulation efficiency, the particles of a granular material are considered as frictional discs in two dimensions or frictional spheres in three dimensions [45]. However, real granular materials are mostly composed of non-spherical granules, with non-smooth surfaces. Therefore, the effect of particle shape is one of the important features that should be taken into account for a realistic description of grain systems.

Azéma and Radjai [46], by using elongated particles in DEM simulation, revealed that the force distribution between the particles becomes significantly broader when the particles become more elongated. Using overlapping spheres in the DEM, Majidi et al. [47] were able to simulate the packing density of coke aggregates with their real shape. Tian et al. [48], by using biaxial discrete element simulation on the specimen with overlapped spheres, showed that the shear band formation and inclination is strongly influenced by the shape of the particles.

In addition, Zhou et al. [49] employed the finite element mesh to generate polygon shape particles in their two-dimensional DEM simulations. They found that the shape and crushability of particles are affected the formation of shear band. All the mentioned attempts to consider the shape of particles in DEM simulations encounter a significant increase in the computational cost. Therefore, they confront serious limitations for the three-dimensional modeling of granular materials in the real size. Hence, it can be beneficial to use methods that implicitly consider the effects of particle shape on the governing relationships between particles.

By carrying out biaxial tests on two-dimensional particles with an elliptical cross-section and taking photoelastic images at different stages during the deformation, Oda et al. [50] concluded that particle rolling is a major mechanism of microscopic deformation. Oda et al. [51], by conducting experiments on several samples of natural sands and using X-ray and optical microscope, deduced that the failure in granular materials not only causes very large cavities inside the shear bands but also creates a high gradient of particle rotation along the shear band boundaries. However, with conventional DEM, the existence of these large voids and high rotation gradients cannot be predicted.

Iwashita and Oda [12] found that, by modifying the conventional DEM and taking into account the rolling resistance between the particles, they could provide a more realistic model that could explain the existence of large voids and high gradients of particle rotation inside the shear bands. It is worth noting that the rolling resistance is defined as a coupled interaction between the grains that resists the rotation of particles [52]. They showed that the formation of a shear band in a granular material is strongly affected by the rolling resistance.

In addition, Oda and Iwashita [52], in their studies on the formation of shear bands in natural sand samples, concluded that the rolling resistance can also be due to the shape of particles. Since the natural particles have different shapes and various surfaces, the rolling resistance can be interpreted as both a modifier of the particle collision model [53] and a criterion of particle shape [54] in DEM modeling.

Moreover, by performing a triaxial test on mono-sized glass beads and simulating using DEM, Wu et al. [55] showed that increasing the rolling resistance leads to an increase in the shear strength. Hazeghian and Soroush [44], by conducting two-dimensional DEM simulation of sand specimens, illustrated the significant role of the rolling resistance in the energy dissipation within shear bands.

Zhao et al. [56] performed a series of three-dimensional discrete element simulations of triaxial compression tests on spherical particles with rolling resistance and elongated particles (super-ellipsoids) and concluded that the rolling resistance model is able to reproduce the main features of the mechanical behavior of the granular material. Moreover, they inferred that the rolling resistance model could consider the surface roughness of particles in discrete element method simulations.

Although the effects of particle shape can be considered with different methods, such as overlapped spheres particle [47] and elongated particles [46], in DEM, the utilization of rolling resistance can lead to a significant reduction in computational cost. Therefore, an in-depth study of the effect of rolling resistance on the behavior of granular materials not only leads to a more practical modeling of the coke aggregates but also considers the effects of particle shape on the type of failure.

By using three-dimensional DEM simulation, this paper investigates the effect of particle size distributions and the rolling resistance as a parameter to consider particle shapes on the failure behavior of coke aggregates during the compaction process. Note that the optimum number of particle for failure analysis and the effect of strain rate and confining pressure on the failure behavior of mono-size coke aggregates are already known from our previous work [5] and are not addressed in this paper.

In Section 2, the concept of the DEM with rolling resistance effect is reviewed and a comparison between the overlapped spheres model and employing the rolling resistance for considering the shape of particles is discussed. In Section 3, the effect of particle size distributions on the mechanical behavior of the coke aggregates, both in the presence and absence of the rolling resistance, is investigated. The influence of the rolling resistance on the mechanical behavior of the coke aggregate is presented in Section 3. In Section 4, the failure analysis is presented based on the second-order work criterion and the failure mode of specimens is discussed. Section 5 discusses the physical interpretation of the particle size distribution and rolling resistance on the failure behavior. Section 6 summarizes and discusses the most salient results of this work.

## 2. Discrete Element Method (DEM) Simulation

DEM was introduced by [45] to simulate the behavior of granular materials in which the particles are considered as an essential component of the mechanical behavior of granular materials. Many attempts have been made to model the behavior of anode paste using DEM [47,57]. Although the modeling of the anode paste has complexities, such as different size distribution, particle shape, solid-fluid interaction, and coal-tar pitch dependence on temperature, it has been shown that DEM can successfully simulate some properties of the anode coke aggregates, such as the bulk density [57], the failure of mono-size coke aggregates [5], and the electrical resistivity [58]. Therefore, DEM has shown that it has many capabilities to provide a realistic model of the coke aggregate samples.

### 2.1. A Summary of DEM

**Figure 1:**
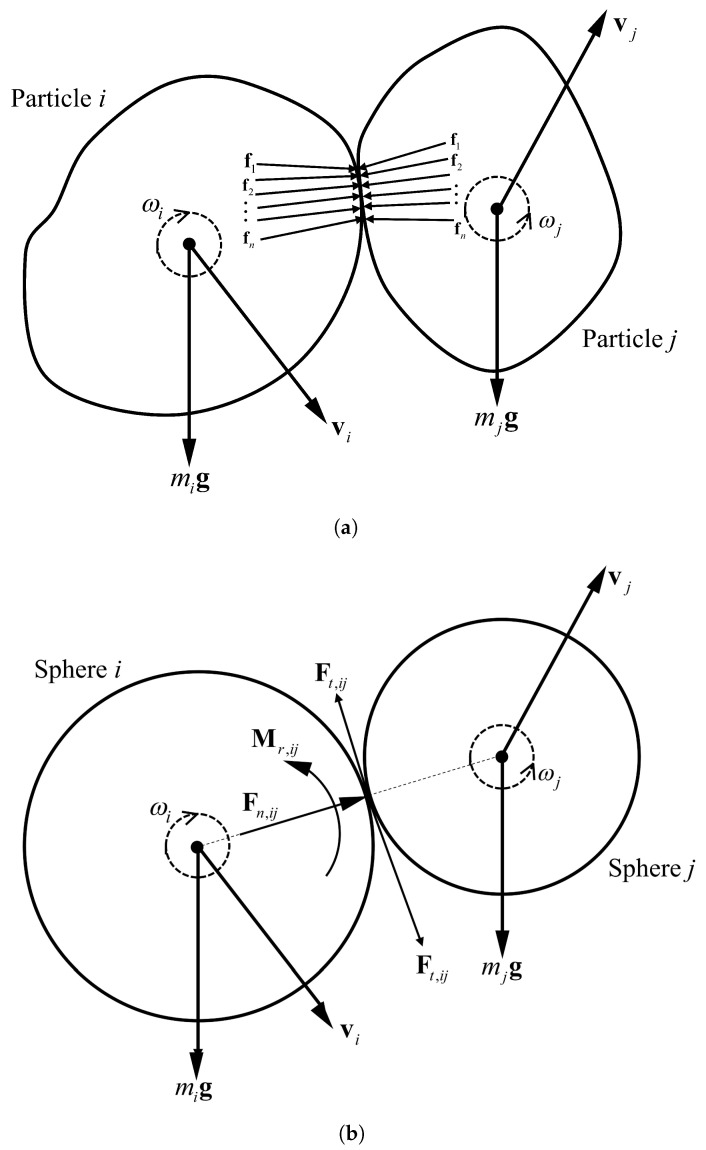
The interaction between (**a**) two real particles, and (**b**) two spheres in DEM ($m_{i}$ is the mass of particle *i*, g is the gravitational acceleration, $F_{t,ij}$ is the tangential contact force between the particles, $F_{n,ij}$ is the normal contact force between the particles, $M_{r,ij}$ is the rolling moment between the particles, $f_{n}$ is the force interaction between the particles, $v_{i}$ is the linear velocity, and $\omega_{i}$ is the angular velocity).

Let us consider two particles with arbitrary shape (Particles *i* and *j* in Figure 1a) that are in contact with each other on a surface and with force interactions between them ($f_{1},f_{2},\cdots ,f_{n}$). Now, if we want to assume these two particles as two spheres with radii $R_{i}$ and $R_{j}$, in order to be able to consider the effects of these force interactions, we can apply a rolling moments ($M_{r,ij}$) at the point of contact of the two spheres in addition to the normal ($F_{n,ij}$) and tangential ($F_{t,ij}$) contact forces. This rolling moment, in addition to replacing the role of force interactions between the particles, can also be a parameter of the shape of the particles that prevents their free rotation. Therefore, the motion of particle *i* is governed by the Newton–Euler equations as follows:

(1) $$\begin{array}{cc} m_{i}g+\sum_{j=1}^{N_{i}}F_{n,ij}+\sum_{j=1}^{N}F_{t,ij} & =m_{i}\frac{dv_{i}}{dt},\\ \sum_{j=1}^{N_{i}}M_{r,ij} & =I_{i}\frac{d\omega_{i}}{dt}, \end{array}$$

where $N_{i}$ is the number of contacts, $m_{i}$ is the particle mass, and $I_{i}$ is the principal moment of inertia. In addition, the normal ($F_{n,ij}$) and tangential ($F_{t,ij}$) contact forces as well as the rolling resistance moment ($M_{r,ij}$) are characterized by:

(2) $$\begin{array}{cc} F_{n} & =K_{n}\delta_{n},\\ \Delta F_{t} & =-K_{t}\Delta U_{t}\;\;\;\mathrm{with}\;\;\;\|F_{t}\left\|\leq \right\|F_{n}\|\tan\phi_{c},\\ \Delta M_{r} & =-K_{r}\Delta \theta_{r}\;\;\;\mathrm{with}\;\;\;\|M_{r}\left\|\leq \right\|F_{n}\|\eta_{r}\min\left(R_{i},R_{j}\right), \end{array}$$

where $K_{n}$, $K_{t}$, and $K_{r}$ are the constant normal stiffness, the constant tangential stiffness, and the constant of rolling stiffness, respectively; $\phi_{c}$ is the contact friction angle; $\eta_{r}$ is the coefficient of rolling friction; $\delta_{n}$ is the overlapping distance between spheres; $U_{t}$ is the relative tangential displacement at the contact point; and $\theta_{r}$ is the relative rolling rotation of particles. The constants of stiffness are defined from a elastic modulus, *E* and dimensionless tangential and rolling coefficients, $\alpha_{t}$ and $\alpha_{r}$, respectively:

(3) $$K_{n}=2E\frac{R_{i}R_{j}}{R_{i}+R_{j}};\;\;K_{t}=\alpha_{t}K_{n};\;\;K_{r}=\alpha_{r}R_{i}R_{j}K_{t}.$$

The rolling resistance model is phenomenologically developed to produce a larger simulated shear strength [56]. In other words, by considering this model, it is possible to simulate the phenomenon that particles have less rotation due to the effect of their shape. Hosn et al. [59] numerically showed that the plastic macroscopic behavior of the granular material is a function of the plastic parameters at the microscopic scale ($\phi_{c}$ and $\eta_{r}$), and depends mainly on the plastic rolling moment ($\|F_{n}\|\eta_{r}\min\left(R_{i},R_{j}\right)$) reflecting the shape of particles. Therefore, the main parameter for considering the particles shape effect is $\eta_{r}$, and the dimensionless rolling coefficient $\alpha_{r}$ does not effect on the plastic macroscopic behavior of the granular materials [59].

### 2.2. Non-Spherical Particle Shape

**Figure 2:**
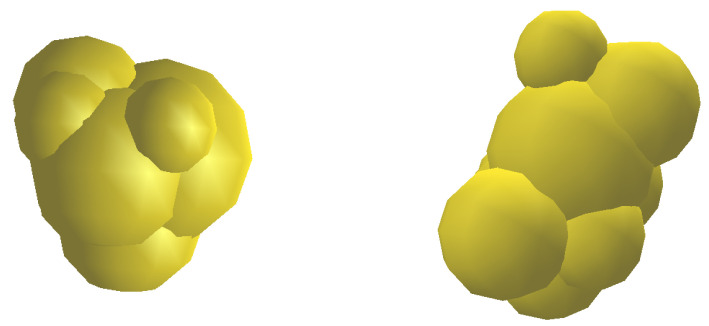
Example of using the overlapped spheres particle model to create the real shape of particles in the discrete element method modeling technique.

As explained in the previous section, one method to consider the effect of particle shape in DEM simulations is to use overlapped spheres particle model. For this reason, 50 coke particles were randomly selected and digitized by a 3D scanner, and then the overlapped spheres were generated from the coke particles by using the Automatic Sphere-clump Generator (ASG) software (Cogency, Cape Town, South Africa). Figure 2 shows two examples of 3D particles generated by overlapping the spheres.

Using the overlapped sphere particles model, similar to the particles used in this paper, Majidi et al. [60] modeled the vibrated bulk density of coke aggregates and showed that their modeling results were in good agreement with the experimental results.

Similar to the real particles, the rotation and contact surface of the overlapped sphere particles produced by this method, is a function of their sphericity. Therefore, comparing the mechanical behavior of DEM simulation of spherical particles with rolling resistance with overlapped sphere particles that have been able to model the density of coke mixture correctly, can have a more accurate understanding of the effects of rolling resistance as a parameter of particle shape on the DEM modeling.

### 2.3. Simulation Setup

In this paper, the DEM computations are performed using the open-source software YADE [61]. The interactions between the particles are simulated in the normal direction to the contact by a linear elastic spring with a stiffness, $K_{n}$, and in the tangential direction by a linear elastic spring with a stiffness, $K_{t}$, and the tangential perfect plasticity with a friction angle $\phi =18^{\circ }$ [57]. The properties of the materials for DEM simulations are also given in Table 1. At the beginning of a computational time-step, the position of all the elements and the boundaries are known.

The contacts are detected by the algorithm according to the known position of the elements and so the magnitude of the possible overlaps between the elements are discovered. The propagated contact forces and momentum on each sphere are then calculated by the interaction law (Equation (1)). Then, the forces are inserted in the law of motion for each particle and the velocity, and the acceleration of the particles are calculated.

The new sphere positions are calculated by applying Newton’s second law of motion. The integration time in Newton’s second law and the interaction contact law are both carried out by way of an explicit scheme. The positions of all the particles and the boundaries in the current time-step are determined by the obtained values. This cycle of the calculations is repeated and solved at each time-step, and thus the flow or the deformation of the material is simulated.

The simulation results presented in this paper were all obtained from two boundary conditions, the periodic and the solid boundary conditions. In the periodic boundary conditions, the particles can go through the boundaries, although the total number of the particles is constant. It is useful for bulk properties modeling, because it ignores the boundary effect on the behavior of the material [62].

Meanwhile, the solid boundary conditions are used for the failure analysis, which is strictly controlled by the boundary effects [63]. Here, it is assumed that the solid boundaries are frictionless. Therefore, the interaction of the particles and the walls is in the normal direction of their contacts. The specimens are generated by randomly inserting grains within a cubic domain (each side is $D_{initial}=10$ cm long) with the possibility of overlap until a target void ratio is achieved. Then, the specimens are left to stabilize.

All the samples are then consolidated to the same initial confining pressure $P_{0}=100$ kPa. Due to the mechanical properties of the samples intended here, their shear responses are examined under a drained conventional triaxial compression loading path. Hence, the load is applied through the displacement-controlled boundaries in the z-direction, while the lateral boundaries are stress-controlled and maintain a constant value for the lateral stresses. Mathematically, stresses and strain rates at boundaries can be expressed as follows:

(4) $$\begin{array}{cc} \dot{\epsilon_{3}}= & 0.05\;s^{-1}\;\;\mathrm{strain}\;\mathrm{controlled}\;\mathrm{in}\;\mathrm{the}\;z\;\left(\mathrm{axial}\right)\;\mathrm{direction},\\ \sigma_{1}=\sigma_{2}= & 100\;\mathrm{kPa}\;\;\mathrm{stress}\;\mathrm{controlled}\;\mathrm{in}\;\mathrm{the}\;x\;\mathrm{and}\;y\;\left(\mathrm{lateral}\right)\;\mathrm{directions}. \end{array}$$

The number of particles in the specimen for failure analysis of the coke aggregates is crucial. Sadeghi et al. [5] showed the optimum number of particles for the mono-size coke aggregates to be around 3000 particles for failure analysis. Therefore, the number of large particles (4–8 US Mesh) in all mixtures is considered to be 3000 and the fine particles (8–14 US Mesh and 14–30 US Mesh) are added to the mixtures according to the typical carbon anode paste formation.

Hence, three different types of mixtures, RVE3000SCM, RVE3000DCM, and RVE3000TCM, were used. RVE3000 represents the representative volume element, which has 3000 large particles, SCM, DCM, and TCM demonstrate the presence of coke particles with different sizes. SCM is a single coke mixture (coke 4–8 US Mesh), DCM is a dual coke mixture (cokes 4–8 US Mesh and 8–14 US Mesh), and TCM is a triple coke mixture (cokes 4–8 US Mesh, 8–14 US Mesh, and 14–30 US Mesh).

**Figure 3:**
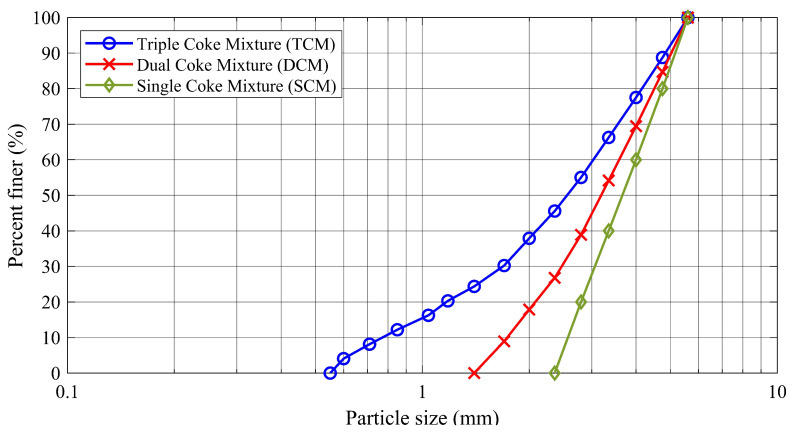
The particle size distributions of coke aggregates used to create specimens.

The number of coke particles in each mixture is shown in Table 2. The ratio of the number of coke particles with different sizes in these mixtures was selected based on the industrial carbon anode production recipe [57]. In addition, the particle size distribution of each mixture is depicted in Figure 3.

### 2.4. Comparison between the Overlapped Spheres Model and Rolling Resistance Model

**Figure 4:**
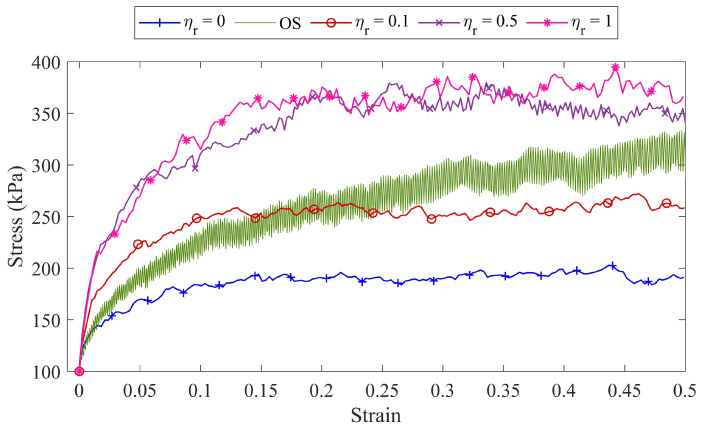
A comparison between the shear behavior of the overlapped spheres particle model (OS) and the rolling resistance model with different coefficients of rolling friction ($\eta_{r}$).

To compare the overlapped sphere particle model and the rolling resistance model, the simulations of the conventional triaxial test ($\sigma_{1}=\sigma_{2}=100$ kPa) are carried out in which the specimen boundaries are periodic. The specimens are created from single coke mixture (SCM) whose particle size distribution is depicted in Figure 3. It should be noted that the number of particles for each sample in this section is 3000. The evolution of axial stress $\sigma_{3}$ versus the axial stress $\epsilon_{3}$ for the samples based on the overlapped sphere particle model (OS) and the roller resistance model with different coefficient of rolling friction ($\eta_{r}$) is shown in Figure 4.

Figure 4 shows that employing the rolling resistance model can reproduce the increasing in the shear strength of the samples caused by the shape of the particles. The axial stress fluctuations in the overlapped sphere particle model shown in Figure 4 are due to the periodic boundary in this simulation. Although, in the overlapped sphere particle method, it is possible to consider the real shape of the particles, due to the fact that coke particles have many different shapes, it is practically not possible to scan all of them. Drawing the Voronoi tessellation for each particle in this model to obtain a micro-strain contour is complicated.

On the other hand, due to the high computational cost of this method, it is not possible to study samples with a large number of particles that are needed to analyze the failure of coke aggregates. Therefore, using the rolling resistance model can be a solution both in terms of reducing computational costs and in terms of the difficulty of drawing Voronoi tessellations.

## 3. Mechanical Behavior of the Coke Aggregates Specimens Along Drained Compression Path

The mechanical behavior of coke aggregate specimens depends on various factors, including the particle size distribution, the particle shape, the confining pressure, and the rate of loading. In a previous work [5], the effects of confining pressure and strain rate on the mechanical behavior of the coke aggregates were investigated, and it was shown that, with increasing the confining pressure from 100 to 250 kPa, the hardening regime and associated axial stress increase. It was also shown that with increasing the axial strain rate from 0.05 to 0.15 $s^{-1}$ (within the static loading range), no significant changes were observed in the mechanical behavior of the coke mixture, except that its hardening regime was reduced.

In this paper, nine different specimens are used to investigate the effects of particle size distribution and particle shape on the mechanical behavior of coke aggregates. Specimens $S_{1}$ to $S_{3}$ are three samples of coke mixtures to study the effects of particle size distribution without considering the rolling resistance. Specimens $S_{4}$ to $S_{6}$ are for revealing the effects of particle size distribution along with rolling resistance. To investigate the effects of the particle shape on the mechanical behavior of the coke aggregates, samples $S_{6}$ to $S_{9}$, made of triple coke mixture (TCM) and with different rolling resistances, were employed.

The detailed characteristics of the specimens are presented in Table 3. The benefit of frictionless solid boundary conditions is to provide the ability to consider the stress at the boundaries as the principal stresses response of the specimens. The initial position of the particles in the specimens is random, and all the specimens are confined with the confining pressure equal to 100 kPa. During the compaction process, the axial strain rate of the specimens is constant and equal to 0.05 $s^{-1}$, while the stresses in the lateral directions are constant and equal to 100 kPa.

### 3.1. Effect of Size Distribution on the Shear Behavior of Coke Aggregates

**Figure 5:**
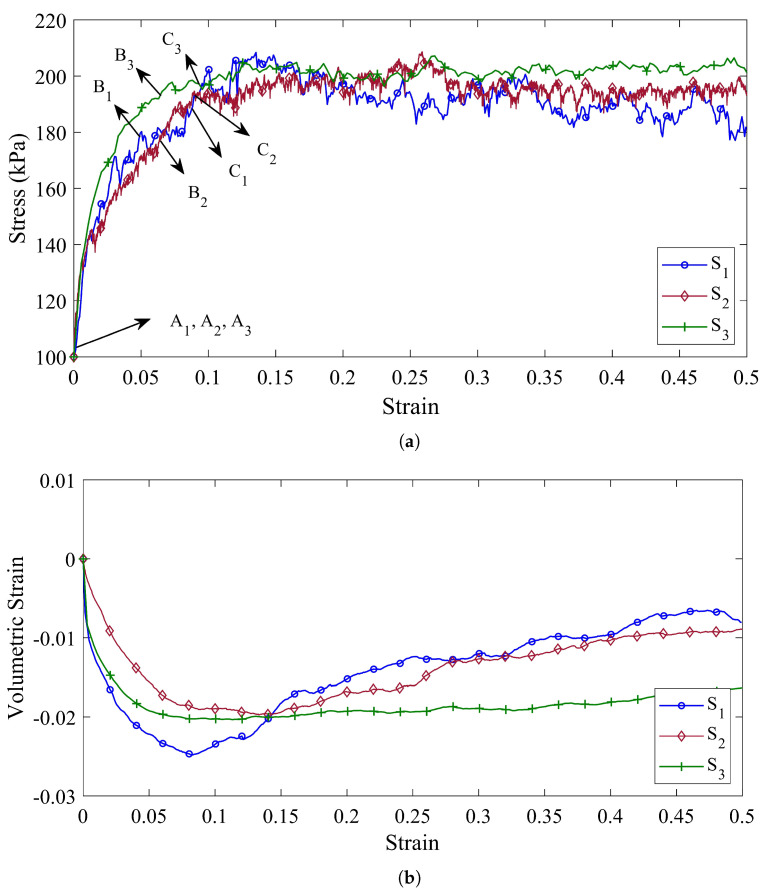
(**a**) The shear stress behavior and (**b**) the volumetric strain behavior of the specimens with different particle size distribution and without the rolling resistance ($\eta_{r}=0$) (Specimen $S_{1}$ is composed of 4–8 US Mesh coke particles, specimen $S_{2}$ is composed of 4–8 US Mesh and 8–14 US Mesh coke particles, and specimen $S_{3}$ is composed of 4–8 US Mesh, 8–14 US Mesh, and 14–30 US Mesh coke particles).

The evolution of the axial stress, $\sigma_{3}$, and the volumetric strain, $\epsilon_{v}$, as a function of the axial strain, $\epsilon_{3}$, for the three specimens $S_{1}$ to $S_{3}$ are shown in Figure 5. The comparison of the mechanical behavior of these three specimens suggests that, although the general behavior of the stress–strain diagram of the three samples is approximately the same, adding the fine particles to the specimens diminishes the fluctuation of the axial stress–strain diagram (Figure 5a).

Therefore, it can be concluded that the shear behavior of the coke aggregates, without considering the effects of particle shape, is mainly a function of the behavior of larger particles. For specimen $S_{1}$, the axial stress increases continuously (positive hardening regime) until it reaches the maximum shear stress $\sigma_{3}=203$ kPa at a strain of $\epsilon_{3}=0.098$. Furthermore, the volumetric strain of specimen $S_{2}$ decreases uniformly to $\epsilon_{v}=0.0247$ at a strain of $\epsilon_{3}=0.082$. From this point on, the slop of the volumetric strain diagram becomes positive and the volume of specimen begins to increase.

In specimen $S_{2}$, where in addition to 4–8 US Mesh coke particles, there are also 8–14 US Mesh coke particles (according to Table 2), the maximum shear stress is reduced to $\sigma_{3}=194$ kPa at a strain of $\epsilon_{3}=0.93$. Moreover, the volumetric strain of specimen $S_{2}$ diminishes continuously to $\epsilon_{v}=0.0194$ at a strain of $\epsilon_{3}=0.14$. Similar to specimen $S_{1}$, the volumetric pressure of specimen $S_{2}$, and consequently its volume also increases after this point.

Although the strain stress diagram of specimen $S_{3}$ increases until it reaches $\sigma_{3}=206$ kPa at strain $\epsilon_{3}=0.116$, its volumetric strain diagram, unlike the previous two specimens, does not increase significantly after reaching its minimum point ($\epsilon_{v}=0.0196$).

On the other hand, due to the fact that the mechanical behavior of granular materials is strongly influenced by the shape of particles, the investigation of the effects of particle size distribution on the mechanical behavior of granular materials without considering the effects of their particle shape will not be comprehensively studied. Therefore, specimens $S_{4}$ to $S_{6}$, in addition to being made of different mixtures of coke, also consider the effects of shape on the mechanical behavior of materials by taking into account the rolling resistance between the particles.

**Figure 6:**
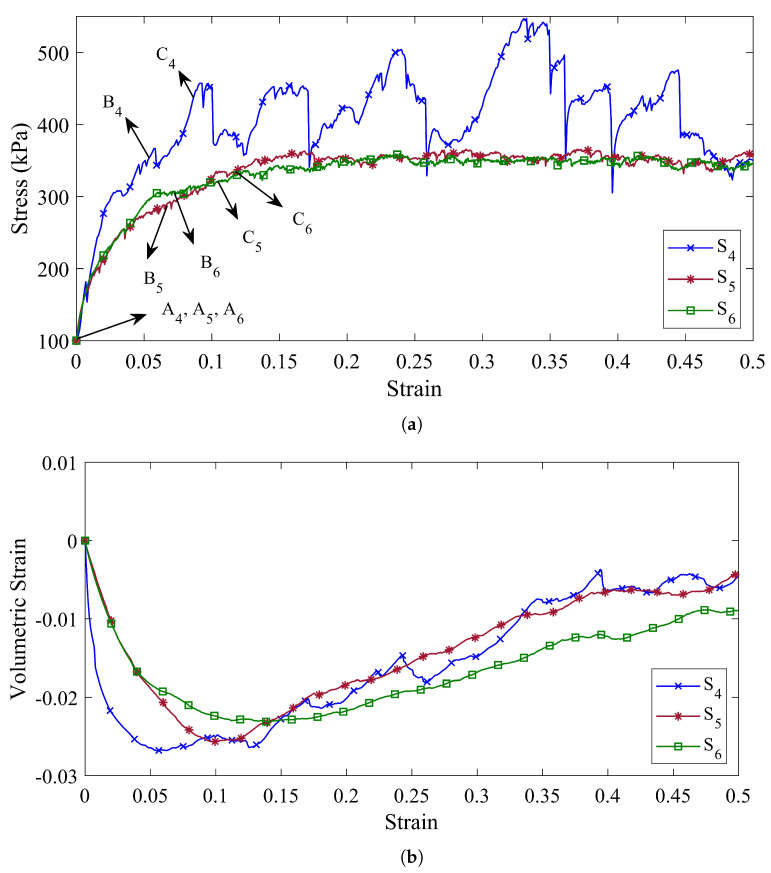
(**a**) The shear stress behavior and (**b**) the volumetric strain behavior of the specimens with different particle size distribution and rolling resistance ($\eta_{r}=0.5$) (Specimen $S_{4}$ is composed of 4–8 US Mesh coke particles, specimen $S_{5}$ is composed of 4–8 US Mesh and 8–14 US Mesh coke particles, and specimen $S_{6}$ is composed of 4–8 US Mesh, 8–14 US Mesh, and 14–30 US Mesh coke particles).

Figure 6a,b represents the shear stress and the volumetric strain versus the axial strain, respectively, for specimens $S_{4}$ to $S_{6}$. Due to the rolling resistance between the particles in these specimens, it is observed that the ability of the specimens to achieve higher stresses increases, and the mechanical behavior of the specimens are more different from each other.

The stress–strain diagram of specimen $S_{4}$ shows that considering the effects of particle shape, the shear behavior of the sample is accompanied by many fluctuations and, at a strain of $\epsilon_{3}=0.091$, it reaches its maximum value ($\sigma_{3}=446$ kPa) for the first time. In addition, its volumetric strain diagram has increased after reaching its minimum value of $\epsilon_{v}=0.028$ at a strain of $\epsilon_{3}=0.057$, although it is accompanied by inconsistencies.

Conversely, the stress–strain diagrams of specimens $S_{5}$ and $S_{6}$ in Figure 6a show that adding smaller particles to the specimen prevents severe fluctuations in its stress–strain diagram. It can also be concluded that, by considering the shape of the particles, the effects of 14–30 US Mesh coke particles on the stress–strain diagram are reduced.

However, the volumetric strain diagrams are not the same for specimens $S_{5}$ and $S_{6}$. Specimen $S_{5}$ reaches its minimum value of $\epsilon_{v}=0.027$ at a strain of $\epsilon_{3}=0.102$ and will then have an upward trend. While the volumetric strain diagram for specimen $S_{6}$ in its strain of $\epsilon_{3}=0.138$ reaches its minimum value of $\epsilon_{v}=0.024$ and then follows an upward trend, albeit with a lower slope than specimen $S_{5}$.

### 3.2. Effect of the Rolling Friction on the Shear Behavior of Coke Aggregates

**Figure 7:**
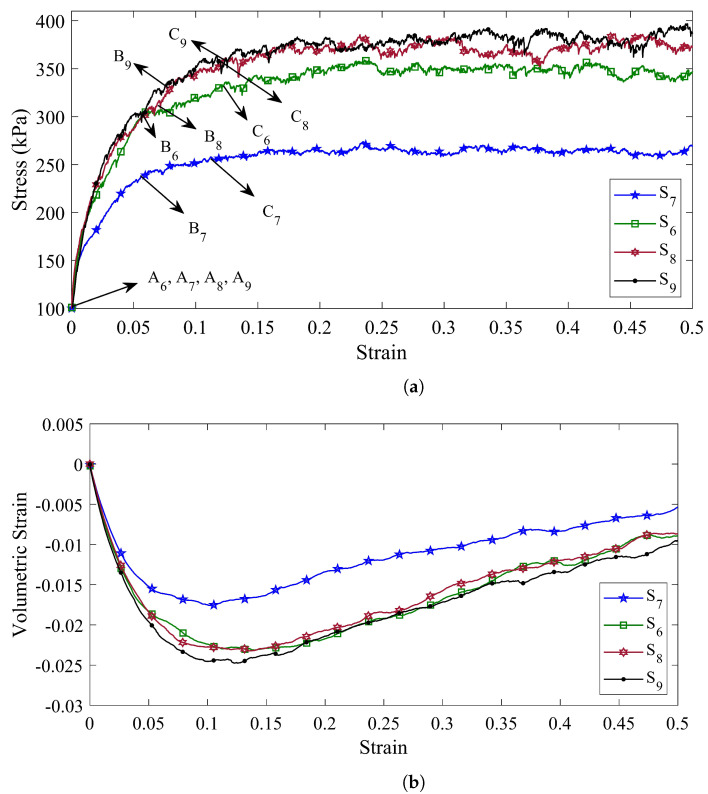
(**a**) The shear stress behavior, and (**b**) the volumetric strain behavior of the specimens with different rolling friction coefficient ($\eta_{r}$) (The rolling friction coefficients for specimens $S_{6}$, $S_{7}$, $S_{8}$, and $S_{9}$ are equal to 0.5, 0.1, 1, and 1.25, respectively).

In the previous section, it was shown that the mechanical behavior of the coke aggregates is strongly dependent on the particle shape parameter. Hence, in this section, the effects of the rolling friction coefficient as a parameter of particle shape on the stress–strain diagram and volumetric strain–strain diagram are also investigated. To achieve this goal, four specimens (specimens $S_{6}$ to $S_{9}$), which are made of a triple mixture of coke (RVE3000TCM), are taken into account. Figure 7a,b represent the axial stress–axial strain and volumetric strain–axial strain, respectively, for the specimens with different rolling friction coefficient ($\eta_{r}$).

According to the previous results, it was found that the rolling resistance model can be considered as a parameter of particle shape in DEM modeling. Therefore, a higher rolling friction coefficient and, consequently, a higher amount of rolling moment can be considered as an indication that the particles have a more complex geometry and move away from the spherical state. According to Figure 7a, by increasing the coefficient of rolling friction, the ability of particles to form stronger force chains increases.

As a result, the specimen’s ability to withstand higher axial stresses increases. For specimen $S_{7}$, the stress–strain diagram reaches its maximum value of $\sigma_{3}=253$ kPa at a strain of $\epsilon_{3}=0.116$. By increasing the coefficient of rolling friction in specimen $S_{6}$, the maximum stress applied to the specimen as well as the amount of strain in the hardening region increases and reaches a stress value of $\sigma_{3}=331$ kPa in the strain of $\epsilon_{3}=0.121$.

Similarly, the maximum amount of stress applied to specimens $S_{8}$ and $S_{8}$ and their maximum amount of strain in the hardening zone are equal to $\sigma_{3}=356$ kPa in the strain of $\epsilon_{3}=0.123$ and $\sigma_{3}=361$ kPa in the strain of $\epsilon_{3}=0.124$, respectively.

The volumetric strain diagram for specimens $S_{6}$ to $S_{9}$ in Figure 7b shows the fact that, by increasing the amount of rolling friction coefficient, the volumetric strain decreases and after reaching its maximum value, it goes through an increasing trend. It should be noted that the slope of the volumetric strain diagram in the ascending trend is almost equal for all four specimens.

## 4. Failure Analysis

### 4.1. Second-Order Work Criterion

In some mechanical problems, by simplifying the conservative and dissipative forces, the existence of a potential energy function can be assumed, and, if this potential function has a strict minimum, stability is achieved. Given the complex mechanism of energy dissipation in the granular materials, a potential energy function cannot be defined for problems with these materials [64]. Therefore, the granular material instabilities cannot be studied through the potential energy function analysis.

In other words, these instabilities are related to the mechanism of inherent deformation of the granular material and do not depend on the potential energy. On the contrary, according to theoretical research, numerical analysis and experimental results, the concept of failure in granular materials can be related to the development of kinetic energy [5,65,66].

In other words, in the failure of granular material, which is accompanied by buckling of force chains, the kinetic energy is suddenly increased and, thus, can be considered as a criterion for the occurrence of failure in the granular materials. In this way, Hill [22] was able to afford a criterion for instability in granular materials in which the control parameters (such as strain or stress at the boundaries) are utilized to delimit the stability of materials.

Accordingly, Hill’s second-order work criterion, as a necessary condition for failure in granular materials, states that a stress–strain state is stable if, for all ($\delta \sigma_{ij}$, $\delta \epsilon_{ij}$) in Eulerian formulation (by assuming small deformations and neglecting geometrical aspects is a quasi static deformation) are linked by the constitutive relation, the associated second-order work is strictly positive [67]:

(5) $$d^{2}W=\underset{V}{\;\int \int \int \;}\delta \sigma_{ij}\;\delta \epsilon_{ij}\;dV>0\;,$$

where $\sigma_{ij}$ is the Cauchy stress tensor, and $\epsilon_{ij}$ is the strain tensor. Thus, according to Hill, a stress–strain state is unstable if there is at least one loading direction that can be pursued in an extremely small manner without any external energy input. Although this condition is not based on thermodynamic principles, it is still a valuable tool for examining potential instabilities [64].

In order to compute the second order work from the macroscopic variables, three stress states, defined by their deviatoric stress ratio $\eta =\left(3\left(\sigma_{3}-\sigma_{1}\right)/\left(\sigma_{1}+\sigma_{2}+\sigma_{3}\right)\right)$, are considered (represented by the points ($A_{n},B_{n},C_{n}$) in Figure 5a, Figure 6a and Figure 7a for specimens $S_{n}$, n=1,⋯,9). These arbitrary stress states are chosen before the maximum stress condition (Mohr–Coulomb condition) is reached.

In particular, $A_{n}$ (n=1,⋯,9) correspond to the isotropic state for each specimen. The strain states, which are specified in Table 4, constitute initial states on which stress probes (as first introduced by [68]) are performed. Due to the frictionless boundaries of specimens and the fact that lateral stresses are kept equal, the stress probe is written as:

(6) $$\Delta \vec{\sigma }=\left\|\Delta \vec{\sigma }\right\|\left(\cos\left(\alpha \right)\vec{e_{1}}+\cos\left(\alpha \right)\vec{e_{2}}+\sin\left(\alpha \right)\vec{e_{3}}\right)\;.$$

By exposing this stress probe to the specimens, the strain response is obtained directly from DEM as:

(7) $$\Delta \vec{\epsilon }=\left(\|\Delta \vec{\epsilon_{1}}\|\right)\vec{e_{1}}+\left(\|\Delta \vec{\epsilon_{2}}\|\right)\vec{e_{2}}+\left(\|\Delta \vec{\epsilon_{3}}\|\right)\vec{e_{3}}\;.$$

**Figure 8:**
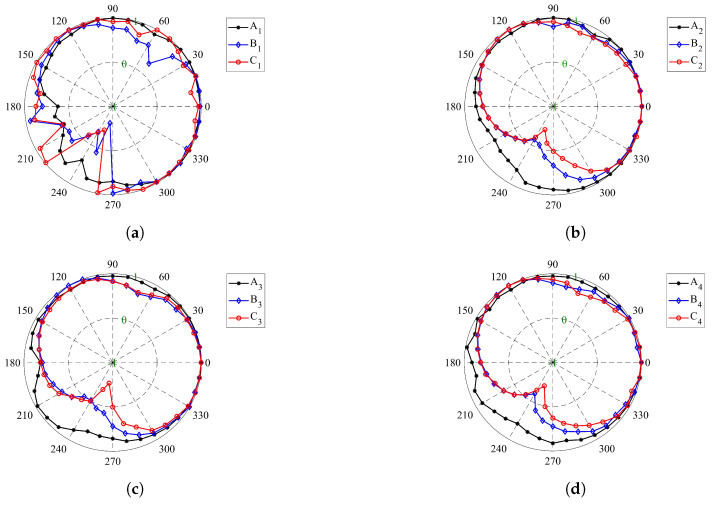

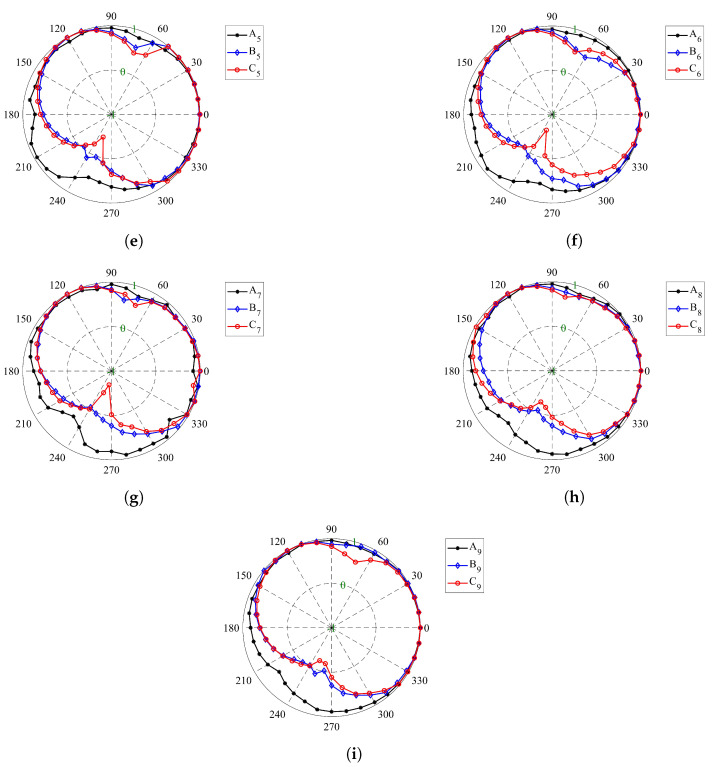
Circular diagrams of the normalized second-order work of (**a**) specimen $S_{1}$, (**b**) specimen $S_{2}$, (**c**) specimen $S_{3}$, (**d**) specimen $S_{4}$, (**e**) specimen $S_{5}$, (**f**) specimen $S_{6}$, (**g**) specimen $S_{7}$, (**h**) specimen $S_{8}$, (**i**) specimen $S_{9}$.

As the stress probe and its strain response are equal in the lateral direction, they could be represented on a two dimensional diagram. Stress probes are performed from an initial stress–strain state by imposing a loading vector $\Delta \vec{\sigma }$ defined in the Rendulic plane of stress increments ($\sqrt{2}\Delta \sigma_{1},\Delta \sigma_{3}$). The norm of $\Delta \vec{\sigma }$ assumed to be 10 kPa. The angle α between the $\sqrt{2}\Delta \sigma_{1}$ and $\Delta \vec{\sigma }$ is increased from $0^{\circ }$ to $360^{\circ }$ by increments of $10^{\circ }$ to check each stress direction. The maximum axial strain rate for applying the stress probe for specimens is equal to 0.05 $s^{-1}$. The corresponding response vectors $\Delta \vec{\epsilon }$, defined in the Rendulic plane of the strain increments ($\sqrt{2}\Delta \epsilon_{1},\Delta \epsilon_{3}$) are computed. Once the strain response $\Delta \vec{\epsilon }$ is computed for each stress probe, by using Equation (5) the macroscopic normalized second-order work is computed as:

(8) $$d^{2}\bar{W}=\frac{\Delta \vec{\sigma }.\Delta \vec{\epsilon }}{\|\Delta \vec{\sigma }\|\|\Delta \vec{\epsilon }\|}\;,$$

for all investigated stress directions and considered strain states. It is worth mentioning that the value of normalized second-order work is in the range of [−1,1]. Figure 8 represents the value of the normalized second-order work for the specimens $S_{n}$ (n=1,⋯,9) at their critical stress state. The dashed circles in Figure 8 demonstrate the zero value for the second-order work. Therefore, when $d^{2}\bar{W}$ is negative the plot is inside the dashed circles, whereas plot is outside the dashed circles for positive values of $d^{2}\bar{W}$.

### 4.2. Effect of Particle Size Distribution on the Second-Order Work Evolution of Coke Aggregates

The second-order work criterion can determine the necessary conditions that lead to failure in the granular materials. For this reason, it can be employed as a tool to ascertain the stability of a stress–strain state. Although Figure 5 shows that the presence of fine particles causes less fluctuation in the stress–strain diagram, and also a wider the particle size distribution leads to less dilation of the specimen, it is not possible to deduce where the specimen fails. Therefore, different points in the stress–strain diagrams have been selected to evaluate the stability of the specimen. The points $A_{1}$, $A_{2}$, and $A_{3}$ represent the specimens $S_{1}$, $S_{2}$, and $S_{3}$ states in the isotropic stress condition, and according to Figure 8a–c, all the specimens in these points have a positive second-order work and therefore stable conditions.

For the specimen $S_{1}$, the cone of the unstable stress directions (inside the dashed circle zone in Figure 8a) are found for $\sigma_{3}=173.5$ kPa when its corresponding α is in the range of [225°, 268°]. In addition, the stress states of point $C_{1}$, in which the tangent of the volumetric strain diagram (Figure 5b) is zero, are unstable when α is in the range of [227°, 254°]. Figure 8b represents the normalized second-order work for specimen $S_{2}$, in which the fine particles (8–14 US Mesh) are added, and it shows that all the stress states associated with point $B_{2}$ are stable. Moreover, the unstable stress is discovered for the $\sigma_{3}=194$ kPa when its corresponding α is in the range of [229°, 269°].

In a similar way, by adding the 14–30 US Mesh particles in specimen $S_{3}$, the cone of the unstable stress directions are found when the axial stress is equal to 196.1 kPa (Figure 8c). The corresponding α for this unstable stress state is in the range of [229°, 231°]. By comparison the range of the unstable α for the points $B_{2}$ and $B_{3}$ reveals that the unstable zone diminishes when the particle size distribution is more extended. However, by analyzing the response of the stress state at the point $C_{3}$, the unstable stress directions are detected when the range of α is [239°, 273°].

At the same time, by supposing that the particles are not perfect spheres and the rolling resistance between the particles can take into account the particle shape, the instability process in the specimens is postponed. Figure 8d–f show the normalized second-order work for specimens $S_{4}$, $S_{5}$, and $S_{6}$, respectively, and all these three specimens are stable in the isotropic stress states ($A_{4}$, $A_{5}$, and $A_{6}$). As shown in Figure 8d, specimen $S_{4}$ has positive second-order work at stress state $B_{4}$, unlike specimen $S_{1}$. However, specimen $S_{4}$ is found to be unstable when the axial stress is equal to 441 kPa. The cone of the unstable stress directions at point $C_{4}$ is in the range of [222°, 261°].

In specimens $S_{5}$ and $S_{6}$, the fine particles played a stabilizing role. Accordingly, not only does the instability not occur at points $B_{5}$ and $B_{6}$ but also specimens $S_{5}$ and $S_{6}$ remained stable until the axial strain of 0.105 and 0.121, respectively. For specimens $S_{5}$ and $S_{6}$, the beginning of the instability occurs at points $C_{5}$ and $C_{6}$, respectively, in which the volumetric strain diagrams for these specimens (Figure 6b) reach their minimum.

The unstable stress directions for these points are detected when the range of α is [224°, 257°] for specimen $S_{5}$ and [229°, 261°] for specimen $S_{6}$. Thus, it was shown that adding fine particles to the specimens could increase the stability of the coke mixture, and the role of fine particles on the stability of the coke mixture becomes more pronounced by taking into account the shape of the particles.

### 4.3. Effect of the Rolling Friction on the Second-Order Work Evolution Of Coke Aggregates

In the previous section, the effect of rolling strength, as a parameter to consider the effects of particle shape, on the coke mixture’s shear behavior was investigated. It was discovered that increasing the shear strength leads to enhance the shear strength of the coke mixtures. Therefore, the examination of the impacts of rolling resistance on the stability of the coke mixture will assist in comprehending the failure behavior of the coke mixture.

As with previous specimens, all four specimens ($S_{6}$ to $S_{9}$) are stable under the isotropic stress states (all the normalized second-order works for stress states $A_{6}$ to $A_{9}$ in the all directions are outside of dashed circle in Figure 8f–i). As the rolling resistance increases, the range in which the coke mixture is stable also increases.

The second-order work response of specimen $S_{6}$ was examined in the previous section. For specimen $S_{7}$, the unstable stress directions are recognized for $\sigma_{3}=258.2$ kPa when its corresponding α is in the range of [238°, 272°]. While the rolling resistance increases in specimens $S_{8}$ and $S_{9}$, the specimens become unstable at higher axial strains. In this case, by analyzing the response of the stress state at the point $C_{8}$, the instability in specimen $S_{8}$ occurs when the axial stress is equal to 352 kPa and the unstable stress directions associated with this stress state are in the range of [236°, 266°].

In a similar way, by increasing the amount of rolling resistance for specimen $S_{9}$, the stress state is unstable when the axial stress reaches 358 kPa (Point $C_{9}$ in Figure 7a). Moreover, the unstable stress direction α corresponding for this stress state, is in the range of [241°, 264°].

Therefore, it can be concluded that with increasing rolling resistance, the compression range of the coke mixture increases without occurring the instability. Since the second-order work criterion does not specify the instability mode of specimens, the micro-strain contours are plotted during the compaction process to identify which type of failure modes (localization or diffusing failure) is happened inside the specimens.

### 4.4. Effect of Size Distribution on the Coke Aggregates Failure Mode

The evidence of failure in the granular system can be observed when the system exceeds the critical stress limit. These evidences occur in the case of strain localization failure in the form of localized bands and unloading areas, while in the case of diffuse failure the strain within the material does not follow a specific pattern [69].

Determination of failure mode in the granular materials, in general, requires special laboratory equipment, such as X-ray tomography. While the discrete element method enables us to numerically observe the evolution of the failure state in a specimen. Therefore, thanks to the micro-strain contours inside the specimens [5], the failure mode of the specimen can be identified according to the stress pressure state at its boundaries. The concept of micro-strain has been fully explained in our previous work [5], and we refrain from re-stating it to shorten the topic.

**Figure 9:**
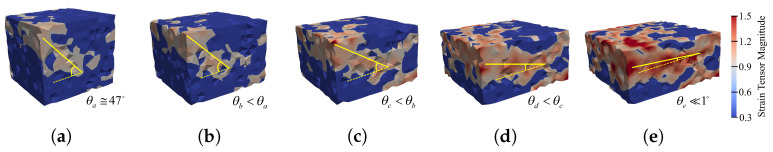
The micro-strain contour evolution during the compaction process of specimen $S_{1}$, (**a**) $\epsilon_{3}=0.1$, (**b**) $\epsilon_{3}=0.2$, (**c**) $\epsilon_{3}=0.3$, (**d**) $\epsilon_{3}=0.4$, (**e**) $\epsilon_{3}=0.5$.

Figure 9 represents the evolution of the micro-strain of specimen $S_{1}$ during the axial compaction and it has been shown that specimen $S_{1}$ fails when the axial stress and the axial strain are equal to 173.5 kPa and 0.0413, respectively. The specimen deformed homogeneously at the beginning of the compaction. However, the deformation field is no longer homogeneous when the compaction increases and micro-strain field localizes into a shear band.

The initial angle between the shear band and the maximum principal stresses plane (here XY-plane) is about $\theta_{a}≅47^{\circ }$. The angle decreases when the compaction increases and at the end of the compaction process, it tends to zero ($\theta_{e}≃0$). Therefore, the shear band turns to a compaction band at the end of the compaction process. This reduction in the angular shear band by increasing the compression process is consistent with the results of Das et al. [70].

By adding smaller particles to the coke mixture in specimen $S_{2}$, the failure behavior of the sample changes. As in the previous specimen, it initially experiences a homogeneous deformation. However, as soon as it exceeds the stability limit, it deforms heterogeneously. As in this specimen, the particles forming the force chain are not able to withstand the bending moment (there is no rolling resistance between the particles), a single deformation band is not observed.

**Figure 10:**
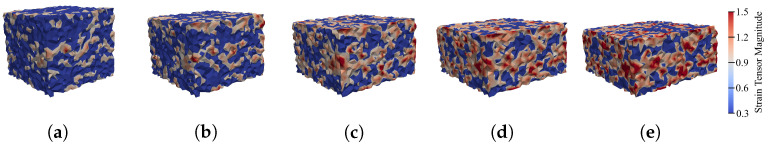
The micro-strain contour evolution during the compaction process of specimen $S_{2}$, (**a**) $\epsilon_{3}=0.1$, (**b**) $\epsilon_{3}=0.2$, (**c**) $\epsilon_{3}=0.3$, (**d**) $\epsilon_{3}=0.4$, (**e**) $\epsilon_{3}=0.5$.

**Figure 11:**
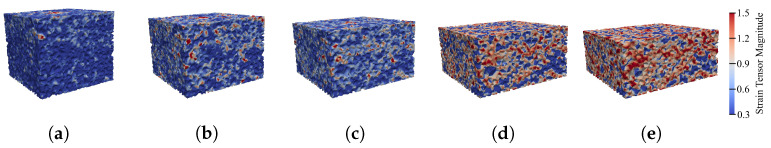
The micro-strain contour evolution during the compaction process of specimen $S_{3}$, (**a**) $\epsilon_{3}=0.1$, (**b**) $\epsilon_{3}=0.2$, (**c**) $\epsilon_{3}=0.3$, (**d**) $\epsilon_{3}=0.4$, (**e**) $\epsilon_{3}=0.5$.

However, it can be inferred from Figure 10 that the failure mode is of the strain localization type. Similarly, the same argument can be executed about how specimen $S_{3}$ behaves in the failure state, except that, in specimen $S_{3}$, the presence of very fine particles (14–30 US Mesh cokes) causes the deformation field in the specimen to be more uniform. Hence, although there is no single observable deformation band, local deformation bands are visible in Figure 11.

The deformation study of specimens $S_{1}$ to $S_{3}$ was performed with the assumption that the particles are spherical and they are in contact with each other at one point. In spite of that, the particles have different shapes and are in contact on one surface instead of a point. Therefore, the study of the effects of particle size dispersion on the fracture state of granular materials is realistic if we can take into account the particle shape characteristics and the complex contact surface between them in modeling.

In Section 2.4, it was shown that the behavior of particles with real shapes can be modeled using the rolling resistance between spherical particles. Iwashita and Oda [12] numerically showed that the DEM modeling can give a realistic description of the formation of shear bands in granular materials when the rolling resistance between the particles is considered. Therefore, in specimens $S_{4}$ to $S_{6}$, the effects of particle size distribution on the failure mode of granular materials are investigated while the particles of these specimens can resist rolling moments.

**Figure 12:**
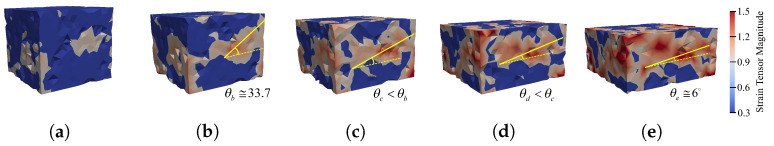
The micro-strain contour evolution during the compaction process of specimen $S_{4}$, (**a**) $\epsilon_{3}=0.1$, (**b**) $\epsilon_{3}=0.2$, (**c**) $\epsilon_{3}=0.3$, (**d**) $\epsilon_{3}=0.4$, (**e**) $\epsilon_{3}=0.5$.

Figure 12 shows the micro-strain contour evolution in specimen $S_{4}$. It initially undergoes an almost uniform deformation. After reaching its stress limit, the stability fails, and this failure manifests itself in the form of localized deformation. Comparing with Figure 9, it can be seen that, by adding the rolling resistance, the stability, and consequently the amount of homogeneous deformation of the sample increases, and the initial angle of the shear band also reduces ($\theta_{b}≅33.7^{\circ }$).

However, at the end of the deformation, the angle of shear band with the maximum principal stresses plane does not reach zero ($\theta_{e}≅6^{\circ }$), and thus the shear band remains in the form of shear band at the end of the compaction process. On the other hand, as in specimens $S_{2}$ and $S_{3}$, the failure mode in specimens $S_{5}$ and $S_{6}$ is of the strain localization type with the difference that, in specimens $S_{5}$ and $S_{6}$, a single shear band is formed.

**Figure 13:**
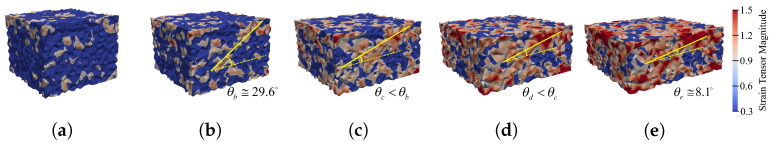
The micro-strain contour evolution during the compaction process of specimen $S_{5}$, (**a**) $\epsilon_{3}=0.1$, (**b**) $\epsilon_{3}=0.2$, (**c**) $\epsilon_{3}=0.3$, (**d**) $\epsilon_{3}=0.4$, (**e**) $\epsilon_{3}=0.5$.

Figure 13 reveals that the homogeneous deformation range for specimen $S_{5}$ increases by adding the rolling resistance to the discrete element method modeling. The initial shear band slope for this sample is $\theta_{b}≅29.6^{\circ }$. As the axial strain increases, the value of this angle decreases and reaches $\theta_{e}≅8.1^{\circ }$ at the end of the compaction process.

**Figure 14:**
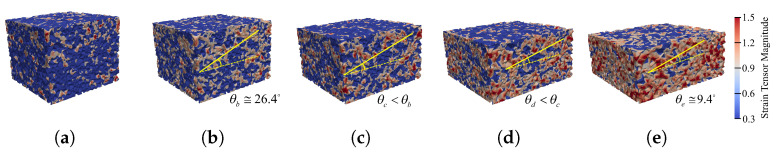
The micro-strain contour evolution during the compaction process of specimen $S_{6}$, (**a**) $\epsilon_{3}=0.1$, (**b**) $\epsilon_{3}=0.2$, (**c**) $\epsilon_{3}=0.3$, (**d**) $\epsilon_{3}=0.4$, (**e**) $\epsilon_{3}=0.5$.

Figure 14 indicates that the initial angle of the shear band in specimen $S_{6}$ is equal to 26.4${}^{\circ }$, and its value at the end of the compaction process reaches about 9.4${}^{\circ }$. By comparing Figure 14 with Figure 12 and Figure 13, it can be inferred that, by adding finer particles to the coke mixture, the initial angle of the shear band with the maximum principal stresses plane decreases, but the angle at the end of the compaction process increases. In other words, by adding fine particles to the specimen, the shear band slope and also its tendency to convert to a compaction band (very small angle $\theta_{e}≅0^{\circ }$) is reduced.

As a consequence, the presence of fine particles in the coke aggregates reduces the probability of the formation of compaction bands in the granular material. Since the compaction bands were introduced by Sadeghi et al. [5] as a possible cause of the horizontal cracks in the stub-hole area in the carbon anodes, recognizing the role of fine particles in the coke mixture can lead to a better understanding of the potential factors that cause horizontal cracks and, thus, improve the performance of carbon anodes in the aluminum reduction cells.

### 4.5. Effect of Rolling Resistance on the Coke Aggregates Failure Mode

The shape of particles is one of the most important parameters affecting the failure behavior of the granular materials. It was also shown that in DEM modeling, the particle shape can be considered by the rolling resistance between spherical particles. Therefore, by increasing the rolling resistance, the particles with less sphericality are modeled. In the previous section, it was shown that, by increasing the rolling resistance between particles, the strength of the specimens against shear loading increases.

The correctness of this statement, i.e., the increase of the stability of the specimen with increasing rolling resistance, was also investigated. Therefore, in this section, the effects of rolling resistance on the failure mode of the coke mixture are investigated. To achieve this goal, specimens $S_{6}$ to $S_{9}$ are considered, for which the rolling resistance was varied by assigning different rolling friction coefficient ($\eta_{r}$) values. The rolling friction coefficient for specimens $S_{6}$, $S_{7}$, $S_{8}$, and $S_{9}$ is equal to 0.5, 0.1, 1, and 1.25, respectively.

**Figure 15:**
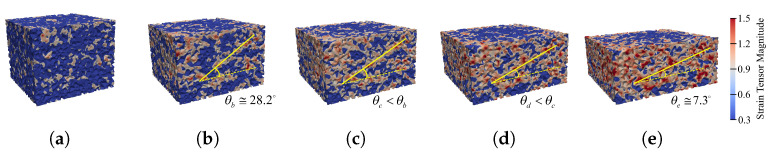
The micro-strain contour evolution during the compaction process of specimen $S_{7}$, (**a**) $\epsilon_{3}=0.1$, (**b**) $\epsilon_{3}=0.2$, (**c**) $\epsilon_{3}=0.3$, (**d**) $\epsilon_{3}=0.4$, (**e**) $\epsilon_{3}=0.5$.

As shown in Figure 15, specimen $S_{7}$ fails locally after exceeding the stability stress limit. The presence of rolling resistance between the particles of this sample causes a single shear band to be formed. The initial angle of this shear band with the maximum principal stresses plane is $\theta_{b}≅28.2^{\circ }$. As the axial strain increases, the value of this angle decreases and reaches $\theta_{e}≅7.3^{\circ }$ at the end of the compaction process. Thus, although the shear band slope decreases with increasing axial strain, its value is still greater than zero. As a result, the shear band remains a shear band at the end of the compaction process, although the amount of compressive stress stored in it increases.

**Figure 16:**
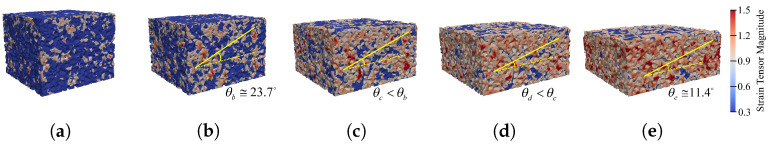
The micro-strain contour evolution during the compaction process of specimen $S_{8}$, (**a**) $\epsilon_{3}=0.1$, (**b**) $\epsilon_{3}=0.2$, (**c**) $\epsilon_{3}=0.3$, (**d**) $\epsilon_{3}=0.4$, (**e**) $\epsilon_{3}=0.5$.

Figure 16 represents the evolution of micro-strain during the compaction process for specimen $S_{8}$. By increasing the amount of the rolling friction coefficient in specimen $S_{8}$, the deformation occurs more homogeneously in the specimen. In addition, by increasing the axial strain and exceeding the stability stress limit, the specimen undergoes a strain localization failure.

**Figure 17:**
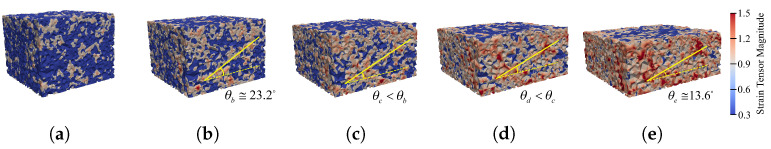
The micro-strain contour evolution during the compaction process of specimen $S_{9}$, (**a**) $\epsilon_{3}=0.1$, (**b**) $\epsilon_{3}=0.2$, (**c**) $\epsilon_{3}=0.3$, (**d**) $\epsilon_{3}=0.4$, (**e**) $\epsilon_{3}=0.5$.

The initial shear band slope in specimen $S_{8}$ is equal to $\theta_{b}≅23.7^{\circ }$, which shows a decrease compared to the initial shear band slope in specimens $S_{6}$ and $S_{7}$. Moreover, during the compaction process, the amount of slope decreases and finally reaches $\theta_{e}≅11.4^{\circ }$. As shown in Figure 17, this trend continues for specimen $S_{9}$.

The initial angle of the shear band with the maximum principal stresses plane is equal to $\theta_{b}≅23.2^{\circ }$ and its final angle of shear band is equal to $\theta_{e}≅13.6^{\circ }$. As a result, by comparing the failure behavior of specimens $S_{6}$ to $S_{9}$ in Figure 14, Figure 15, Figure 16 and Figure 17, it can be concluded that, by increasing the rolling resistance, which indicates a decrease in the sphericality of the particles, the initial angle of the shear band with the maximum stress plane decreases. Conversely, with increasing rolling resistance, the final slope of the shear band increases.

Considering that the slope of the shear band has an inverse relationship with the amount of compressive stress in it (the lower the slope of the shear band, the higher the amount of compressive stress), it can be concluded that increasing the rolling resistance reduces the amount of compressive stress within the shear band.

For this reason, if the rolling resistance between the particles of the granular material increases (the sphericality of the particles decreases) the chance of forming a compaction band in the granular material will be less. This conclusion is valuable because the formation of compression bands in the coke mixture was cited as a possible cause of horizontal cracks in the carbon anodes [5]. Therefore, the use of coke particles with complex geometries (less sphericity) can be effective in reducing the chance of horizontal crack formations in the carbon anodes.

## 5. Discussion

**Figure 18:**
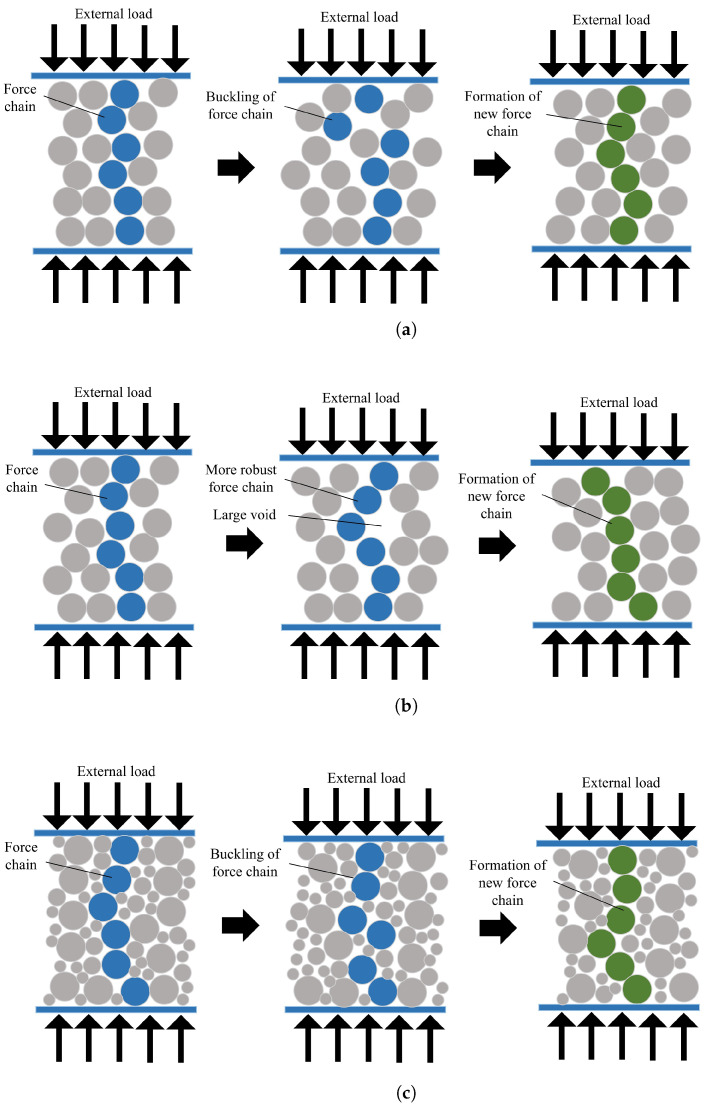
Deformation mechanism of a granular material (**a**) without rolling resistance and small particles, (**b**) with rolling resistance, and (**c**) with small particles.

The effects of particle size distribution and rolling resistance, as a parameter to consider the real particle shape, on the mechanical behavior, the stability, and the failure mode of the coke aggregates were investigated. It was shown that, by adding smaller particles and by increasing the rolling resistance, the possibility of the formation of compaction bands in the specimens decreases. In Figure 18, the reasons for these effects are schematically demonstrated from a physical point of view. Figure 18a shows the deformation of a coke mixture without considering the fine particles and rolling resistance. As the axial deformation increases, the force chains buckle and the specimen fails. As a result of the failure of the mixture, a new force chain with more lateral pressure is formed to provide the necessary resistance to withstand external loads.

Consider a coke mixture with the rolling resistance between its particles as shown in Figure 18b. When the rolling resistance increases, it means that the particles have a more asymmetric geometry, and hence the contact surface between the particles increases. Therefore, increasing the rolling resistance increases the moment applied to each particle. In such a case, the force chain formed between the particles has a higher strength and is more resistant to buckling.

For this reason, the specimen fails at higher strains, and, as a result, the initial shear band angle with the maximum principal stress plane must be reduced. On the other hand, due to increase in the strength of the force chains, the variations in the shear band angle relative to the axial strain decreases. Therefore, increasing the rolling resistance leads to enhancing the final angle of the shear band with the maximum principal stress plane.

Figure 18c represents a coke mixture with fine particles. By adding smaller particles to the specimens, the lateral support of the force chains increases. In this case, by buckling the force chain, the smaller particles quickly fill the existing force gap and prevent the sharp fluctuations of stresses that are felt at the boundaries.

With increasing lateral support of the force chains, the initial shear band angle with the maximum principal stress plane decreases. Moreover, adding fine particles to the coke mixture, reduces the variations in the shear band angle, accordingly at the end of the compaction process, the final angle of the shear band with the maximum principal stresses plane increases. More precisely, when the specimen has a larger particle size distribution, it has a greater tendency to shear band maintain the shear stress within the local area.

## 6. Conclusions

In this work, the failure behavior of coke aggregates and the effects of particle size distributions and shapes were investigated using the discrete element method modeling technique. The failure of granular material is the most important factor in causing defects such as cracks. Therefore, modeling the failure behavior of coke aggregates can identify potential factors for the generation of cracks, specially horizontal cracks, in the carbon anodes. The main conclusions can be summarized as follows:

- Since the anode paste contains the coke particles with different size distributions, the effects of the particle size distribution on the failure behavior of the coke mixture was investigated. The results showed that the mechanical behavior of the coke mixture is a function of the behavior of the larger particles. By adding fine particles to the coke mixture, the fluctuations of the stress–strain diagram of the coke mixture are reduced. In addition, the presence of fine particles in the coke aggregates reduces the tendency of the mixture to dilate.
- It was shown that the mechanical behavior of the coke mixture is strongly dependent on the particle shape. Therefore, the mechanical behavior of the coke mixture in which the particles had their real shape was investigated and it was demonstrated that the rolling resistance can be used as a parameter representing the shape of the particles in DEM modeling. The study of the mechanical behavior of coke mixture for different values of rolling friction coefficients was performed and it was revealed that with increasing rolling resistance between the particles, the tolerance of coke mixture against shear load increases.
- The second-order work criterion was used to find the failure threshold in the specimens. It was shown that, by adding fine particles to the coke mixture, the stability threshold of the mixture increases. In other words, the specimen becomes unstable in the higher axial strains and, as a result, the safe compaction range of the coke mixture (in which the sample does not fail) increases.
- Examination of the second-order work criterion for specimens with different values of rolling friction coefficient showed that, by increasing the rolling resistance, the stability threshold for the specimens increases, and consequently they fail at higher axial strains.
- The mode of failure in the DEM simulation of the coke mixture was determined by micro-strain contour analysis during the compression process. In the absence of rolling resistance between the particles, the results showed that the addition of smaller particles to the coke mixture did not form a single shear band in the sample. This highlights the importance of using the rolling resistance model in DEM modeling to investigate the failure of granular materials.
- It was shown that adding fine particles, while there is rolling resistance between the particles, leads to a single shear band in the specimen. The angle that this shear band adopts with the maximum principal stresses plane decreases during the compaction process. With the addition of smaller particles, the slope of the shear band decreases at the beginning of the compaction process, but the final angle of the shear band increases. In other words, adding fine particles to the coke mixture reduces the possibility of a compaction band at the end of the compaction process. As a result, the possibility of the horizontal cracks caused by compression bands is reduced.
- While the rolling resistance between the particles increases, the shear band angle with the maximum principal stresses plane at the beginning of the compaction process decreases. However, its value at the end of compaction process increases. In this case, it can be concluded that increasing the rolling resistance (using less spherical particles) leads to a reduction in the likelihood of the formation of a compaction band in the coke aggregates. Therefore, the use of coke particles with complex geometries can be considered as a preventive solution for horizontal cracks in the carbon anodes.

The main focus of this article is on the study of the failure behavior of the dry coke aggregates. However, the carbon anode paste is very complex because it is composed of coke aggregates with very wide size distributions and also contains coal-tar pitch, which acts like a viscous fluid during the compaction process. In future research, the role of coal-tar pitch on the failure of the coke aggregates will be explored by using DEM simulation.

## Figures and Tables

**Table 1:** The characteristics of the coke particles used in the discrete element method (DEM) model [57].

Property	US Mesh 4–8	US Mesh 8–14	US Mesh 14–30
Density (kg/$m^{3}$)	1377	1523	1523
Elastic modulus (MPa)	681	681	681
Poisson ratio	0.3	0.3	0.3
Friction angle (rad)	0.31	0.31	0.31
Damping ratio	0.4	0.4	0.4
Dimensionless of tangential coefficient	0.385	0.385	0.385
Dimensionless of rolling coefficient	2	2	2

**Table 2:** The number and distribution of particles in the specimens.

Name of Mixture	4–8 US Mesh ($d_{50}$ = 3.7 mm)	8–14 US Mesh ($d_{50}$ = 1.7 mm)	14–30 US Mesh ($d_{50}$ = 0.8 mm)	Total Number of Particle
RVE3000SCM	3000	0	0	3000
RVE3000DCM	3000	8202	0	11,202
RVE3000TCM	3000	8202	74,910	86,112

**Table 3:** The characteristic of the specimens used to investigate the effects of particle size distribution and rolling resistance on the mechanical behavior of coke aggregates.

Name of Specimens	Mixture	Coefficient of Rolling Friction $\left(\eta_{r}\right)$	Confining Pressure (kPa)	Axial Strain Rate ($s^{-1}$)	Initial Porosity ($\phi_{\mathrm{initial}}$)
$S_{1}$	RVE3000SCM	0	100	0.05	0.468
$S_{2}$	RVE3000DCM	0	100	0.05	0.43
$S_{3}$	RVE3000TCM	0	100	0.05	0.375
$S_{4}$	RVE3000SCM	0.5	100	0.05	0.484
$S_{5}$	RVE3000DCM	0.5	100	0.05	0.45
$S_{6}$	RVE3000TCM	0.5	100	0.05	0.375
$S_{7}$	RVE3000TCM	0.1	100	0.05	0.375
$S_{8}$	RVE3000TCM	1	100	0.05	0.375
$S_{9}$	RVE3000TCM	1.25	100	0.05	0.375

**Table 4:** Deviatoric stress ratio η and axial strain $\epsilon_{3}$ corresponding to the critical points of specimens $S_{n}$ (n=1,2,⋯,9).

	$A_{n}$		$B_{n}$		$C_{n}$	
	$\epsilon_{3}$	η	$\epsilon_{3}$	η	$\epsilon_{3}$	η
$S_{1}$	0	0	0.053	0.616	0.083	0.688
$S_{2}$	0	0	0.054	0.617	0.087	0.714
$S_{3}$	0	0	0.055	0.704	0.088	0.723
$S_{4}$	0	0	0.055	1.391	0.089	1.597
$S_{5}$	0	0	0.059	1.166	0.105	1.269
$S_{6}$	0	0	0.061	1.225	0.121	1.325
$S_{7}$	0	0	0.006	0.946	0.112	1.03
$S_{8}$	0	0	0.064	0.249	0.123	1.383
$S_{9}$	0	0	0.067	0.319	0.124	1.4

## Data Availability

No new data were created or analyzed in this study. Data sharing is not applicable to this article.

## Nomenclature

$\alpha_{r}$	dimensionless rolling coefficient
$\alpha_{t}$	dimensionless tangential coefficient
$\dot{\epsilon }$	strain rate tensor (1/s)
ϵ	strain tensor
σ	Cauchy stress tensor (Pa)
$\delta_{n}$	normal overlapping displacement of particle (m)
$\eta_{r}$	coefficient of rolling friction
$F_{n,ij}$	normal contact force between spheres *i* and *j* (N)
$f_{n}$	force interaction between particles(N)
$F_{t,ij}$	tangential contact force between spheres *i* and *j* (N)
g	the gravitational acceleration (m/s${}^{2}$)
$M_{r,ij}$	rolling moment between spheres *i* and *j* (Nm)
$v_{i}$	linear velocity of particle *i* (m/s)
$\omega_{i}$	angular velocity of particle *i* (rad/s)
ϕ	porosity
$\phi_{c}$	contact friction angle (°)
$\theta_{i}$	the angle between the localized band and the maximum principal stress plane (°)
$\theta_{r}$	relative rolling rotation of particle (rad)
$d^{2}\bar{W}$	normalized second-order work
$d^{2}W$	second-order work (J)
$d_{50}$	the average diameter of particle (m)
$D_{initial}$	the initial length of specimen (m)
*E*	elastic modulus of particle (MPa)
$I_{i}$	principal inertia moment of particle *i* (kg$m^{2}$)
$K_{n}$	normal spring stiffness (N/m)
$K_{r}$	rolling spring stiffness (N/rad)
$K_{t}$	tangential spring stiffness (N/m)
$m_{i}$	mass of particle *i* (kg)
$R_{i}$	Radii of particle *i* (m)
$U_{t}$	relative tangential displacement of particle (m)
